# Strong off-target antibody reactivity to malarial antigens induced by RTS,S/AS01E vaccination is associated with protection

**DOI:** 10.1172/jci.insight.158030

**Published:** 2022-05-23

**Authors:** Dídac Macià, Joseph J. Campo, Gemma Moncunill, Chenjerai Jairoce, Augusto J. Nhabomba, Maximilian Mpina, Hermann Sorgho, David Dosoo, Ousmane Traore, Kwadwo Asamoah Kusi, Nana Aba Williams, Amit Oberai, Arlo Randall, Hèctor Sanz, Clarissa Valim, Kwaku Poku Asante, Seth Owusu-Agyei, Halidou Tinto, Selidji Todagbe Agnandji, Simon Kariuki, Ben Gyan, Claudia Daubenberger, Benjamin Mordmüller, Paula Petrone, Carlota Dobaño

**Affiliations:** 1 ISGlobal, Hospital Clínic, Universitat de Barcelona, Barcelona, Catalonia, Spain.; 2 Antigen Discovery, Inc (ADI), Irvine, California, USA.; 3 CIBER de Enfermedades Infecciosas (CIBERINFEC), Barcelona, Spain.; 4 Manhiça Health Research Center (CISM), Maputo, Mozambique.; 5 Ifakara Health Institute, Bagamoyo Research and Training Centre, Bagamoyo, Tanzania.; 6 Swiss Tropical and Public Health Institute, Basel, Switzerland.; 7 University of Basel, Basel, Switzerland.; 8 Nanoro Clinical Research Unit, Health Science Research Institute, Nanoro, Burkina Faso.; 9 Kintampo Health Research Centre, Kintampo, Brong-Ahafo, Ghana.; 10 Department of Electron Microscopy & Histopathology, Noguchi Memorial Institute for Medical Research, College of Health Sciences, University of Ghana, Legon, Ghana.; 11 Department of Immunology and Infectious Diseases, Harvard T.H. Chen School of Public Health, Boston, Massachusetts, USA.; 12 Lambaréné Medical Research Center (CERMEL), Lambaréné, Gabon.; 13 Kenya Medical Research Institute/Centre for Global Health, Kisumu, Siaya, Nairobi, Kenya.; 14 Noguchi Memorial Institute for Medical Research, University of Ghana, Legon, Ghana.; 15 Department of Medical Microbiology, Radboud University Medical Center, Nijmegen, Netherlands.; 16 Institute of Tropical Medicine and German Center for Infection Research, University of Tübingen, Tübingen, Germany.

**Keywords:** Immunology, Infectious disease, Adaptive immunity, Antigen, Epidemiology

## Abstract

The RTS,S/AS01E vaccine targets the circumsporozoite protein (CSP) of the *Plasmodium falciparum* (*P*. *falciparum*) parasite. Protein microarrays were used to measure levels of IgG against 1000 *P*. *falciparum* antigens in 2138 infants (age 6–12 weeks) and children (age 5–17 months) from 6 African sites of the phase III trial, sampled before and at 4 longitudinal visits after vaccination. One month postvaccination, IgG responses to 17% of all probed antigens showed differences between RTS,S/AS01E and comparator vaccination groups, whereas no prevaccination differences were found. A small subset of antigens presented IgG levels reaching 4- to 8-fold increases in the RTS,S/AS01E group, comparable in magnitude to anti-CSP IgG levels (~11-fold increase). They were strongly cross-correlated and correlated with anti-CSP levels, waning similarly over time and reincreasing with the booster dose. Such an intriguing phenomenon may be due to cross-reactivity of anti-CSP antibodies with these antigens. RTS,S/AS01E vaccinees with strong off-target IgG responses had an estimated lower clinical malaria incidence after adjusting for age group, site, and postvaccination anti-CSP levels. RTS,S/AS01E-induced IgG may bind strongly not only to CSP, but also to unrelated malaria antigens, and this seems to either confer, or at least be a marker of, increased protection from clinical malaria.

## Introduction

The recombinant protein in adjuvant subunit vaccine RTS,S/AS01E (Mosquirix) is the first to be recommended by WHO for use in African children to prevent malaria, following the completion of a phase III trial for licensure and a pilot implementation study (1). This vaccine targets *Plasmodium falciparum* (*P*. *falciparum*), the deadliest of malaria species in humans, a protozoan parasite expressing over 5300 proteins, of which hundreds are located near the parasite surface at some stage of its life cycle, thereby presenting numerous targets for human host antibodies (Abs). The vaccine contains a part of the circumsporozoite (CSP) sequence, a crucial protein on the sporozoite surface that targets *P*. *falciparum* parasites before they reach the liver, and it is coexpressed with hepatitis B surface antigen (HBsAg). Vaccine efficacy (VE) was assessed in the phase III trial between 2009 and 2014 in 11 sites in Africa, yielding estimates of 55.8% in children (2) and 31.3% in infants over 1 year of follow-up (3) yet rapidly decreasing in the following years (4). Our present study is a nested immunology study within the phase III clinical trial.

Immunogenicity studies have established a dose-response relationship between postvaccination anti-CSP Ab levels and VE across multiple trial sites (5, 6), with Ab waning curves over succeeding months closely coinciding with VE decline. Likewise, in other subunit vaccines, circulating Ab levels against the target protein are frequently the best measurable correlate of VE. By contrast, monitoring Ab responses to proteins from the same pathogen that are not included in the vaccine (off-target) is less common, and, when pursued, the objective usually resides in describing the complex interplay of vaccine-induced and naturally acquired immunity against the same pathogen (7, 8). After RTS,S/AS01E vaccination in endemic settings, a number of off-target Ab levels, mainly markers of exposure, were found to be lower in RTS,S/AS01E vaccinees in the months following vaccination (7, 9, 10). These results indicate that acquisition of natural immunity is reduced, or more probably delayed (11), as a consequence of the partial protection conferred by the vaccine.

Unexpectedly, a small group of vaccine off-target Abs has also been found that appeared to be increased in vaccinated children. In a panel of more than 800 proteins tested in samples taken 6 months after primary vaccination from a Mozambican phase IIb trial, a large number of Abs showed expected decreases in RTS,S/AS01E vaccinees, but a much smaller number of Abs were increased, which became a majority when the comparison was restricted to vaccinees with no reported clinical malaria cases during follow-up (7). More recently, a panel of 40 proteins probed in plasmas sampled 1 month after vaccination from the phase III clinical trial showed a small group of antigens (Ags) with increased Ab levels in the RTS,S group (8, 12). More surprisingly, some of these off-target Ab increases induced only 1 month following vaccination were associated with greater protection. In light of this, we then speculated that asymptomatic malaria infections could still take place in RTS,S vaccinees and that these low-level infections could account for an accelerated acquisition of natural immunity. We also considered the possibility of Ab cross-reactivity even when no clear sequence similarities were found.

In vaccinology, cross-reactivity has mainly been studied between key Ags from different pathogen strains or species (heterologous or between-pathogen cross-reactivity), often motivated by the cross-protection that may result thereof (13–15). By contrast, little is known about cross-reactivity against different Ags expressed in the same organism (within-pathogen cross-reactivity) and the potential reinforced protection that may result. However, evidence exists that a degree of cross-reactivity against epitopes of different malarial proteins expressed at different life cycle stages is possible (16, 17) and seems to be mainly driven by immunodominant low-complexity repeat structures characteristic of the malaria parasite, such as those in CSP (18, 19). The advent of high-throughput immunology, capable of screening a specific Ab response against nearly all Ags expressed in the same pathogen, will likely challenge assumptions of within-pathogen cross-reactivity.

The aim of this study was to address the phenomenon of vaccine-induced off-target Ab reactivity occurring immediately after vaccination and through long-term follow-up with booster administration using an unbiased and comprehensive panel of Ags. We used this rich data set to identify direct causal effects of RTS,S/AS01E vaccination on off-target Abs, which were expected to be highest after vaccination and wane over follow-up months similar to anti-CSP Ab levels, as opposed to indirect effects mediated by differential natural acquired immunity, which were expected to build up over time and be negatively associated with vaccination due to reduced parasite exposure.

Our analyses of vaccine off-target Ab reactivity covered a panel of 1000 *P*. *falciparum* antigenic proteins or fragments representing 762 unique protein-coding genes (14% of the 3D7 strain proteome) and allowed us to assess at a large scale whether RTS,S/AS01E-induced off-target Ab reactivity to different Ags is independent of one another or is organized in signatures. We also investigated the association of vaccine off-target Ab reactivity with age, exposure, and other epidemiologic variables. Finally, we assessed whether the intensity of off-target Ab responses immediately following vaccination is a correlate of VE even after adjusting for anti-CSP levels.

## Results

### IgG against many P. falciparum proteins are altered after primary vaccination, with a small group of proteins showing high increases.

**Figure 1:**
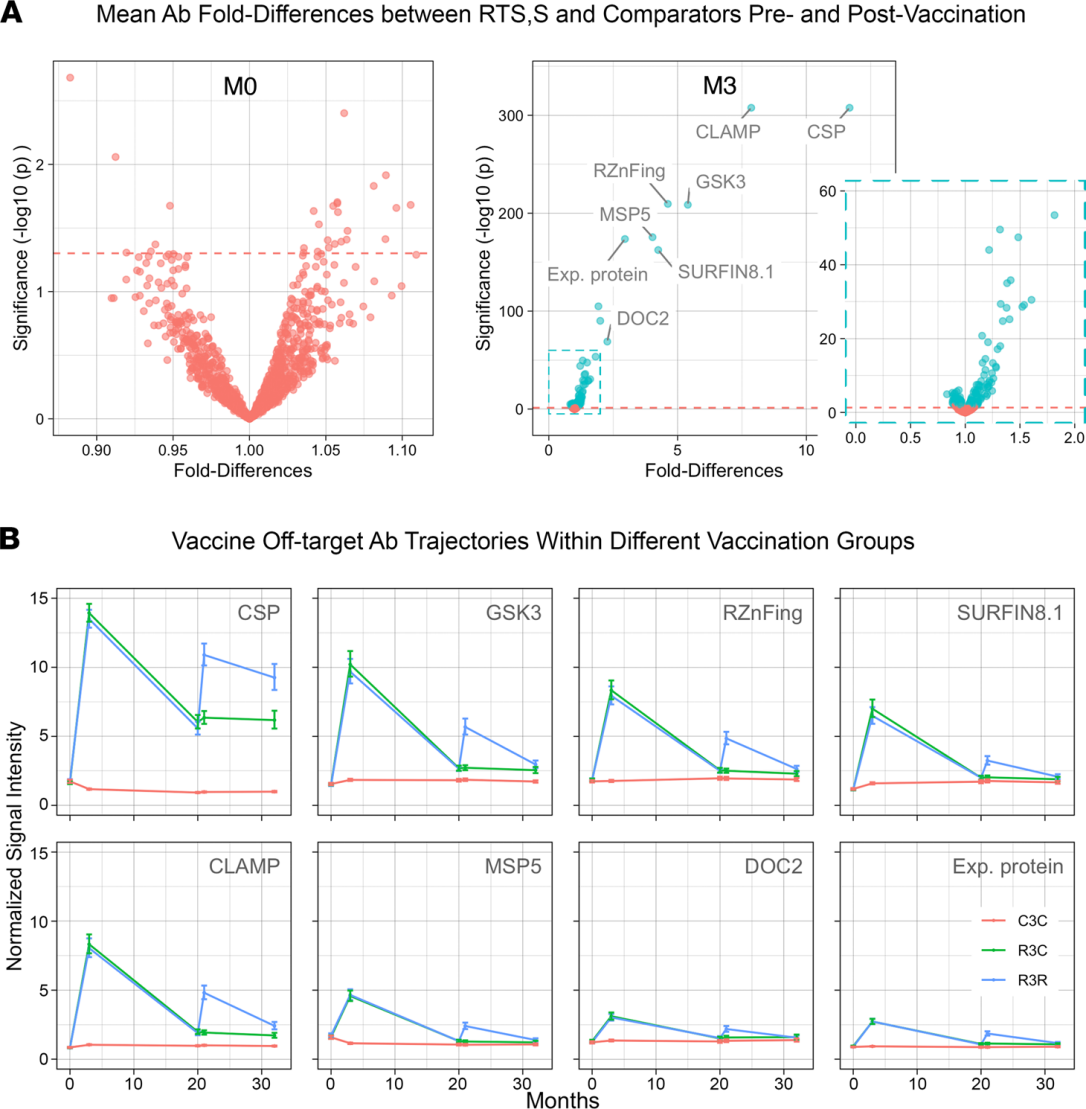
Differential Ab levels across time points. (**A**) Volcano plots from repeated univariate regression models comparing Ab-normalized signal intensity geometric means against 1000 malarial Ags between comparator and RTS,S/AS01E groups, adjusting for age group, site, and sex. Effect sizes in the volcano plots are represented as fold differences (*x* axis) of RTS,S/AS01E over comparators. Green dots correspond to false discovery rate–corrected significant differences; red dots do not. Left: prior to vaccination (M0) when no differences were detected; Right: shortly following vaccination (M3) when more than a hundred Ags were found with significant differential Ab levels. (**B**) Longitudinal trajectories of geometric mean normalized signal intensity and their 95% CI. Panels include the 7 most reactive off-target Abs at M3 in addition to CSP. Blue corresponds to RTS,S vaccination with boost at study month 20 (R3R), green to without boost (R3C), and red to comparator vaccination (C3C).

Prior to vaccination (study month 0, M0), Ab levels exhibited no significant differences to any probed Ags in the microarray between the RTS,S and comparator groups (Figure 1A, left). This result indicated that the study subsample preserved the randomization of the clinical trial vaccination groups and that there were no differences in baseline immunological profiles prior to intervention. Only a month after the primary vaccination concluded (M3), there were significant differences between vaccination groups in 17% of all probed Ags (Figure 1A, right). Some of these Ags elicited surprisingly strong mean Ab-level increases, approaching that of CSP (11.5-fold increase in the RTS,S over the comparator group, CI [11.0–12.3]). Ranking them in decreasing order and only including the largest increases (>2-fold), we encountered a claudin-like apicomplexan microneme protein (CLAMP, 7.8-fold increase, CI [7.2–8.5]), a glycogen synthase kinase (GSK3, 5.4, CI [4.9–5.9]), a RING zinc finger protein (RZnFing, 4.6, CI [4.2–5.0]), the merozoite surface protein 5 (MSP5, 4.0, CI [3.7–4.4]), an exported protein of unknown function (2.9, CI [2.7–3.2]), and a double C2-like domain-containing protein (DOC2, 2.3, CI [2.1–2.5]).

Further significant differences rapidly decreased in magnitude: 6 Ags had geometric mean Ab levels increased in RTS,S vaccinees of 50%–100% over comparators, 14 Ags between 25% and 50%, and as many as 143 Ags with smaller differences (<25%). Of note, 70 Ags from the latter group were not increases but decreases in the RTS,S group (green dots to the left of 1 in the right volcano plot, Figure 1A). Supplemental Table 1 in Supplemental Data 1 (supplemental material available online with this article; https://doi.org/10.1172/jci.insight.158030DS1) contains details of the corresponding fold differences between vaccination groups at M3, as well as at subsequent time points for all Ags probed in the microarray. For validation of results, a purified recombinant MSP5 protein, one of the main off-target Ags, was available and was tested in an orthologous Ab binding assay using quantitative suspension array technology (qSAT) on a subset of samples from this same phase III trial, as well as samples from an independent phase IIb clinical trial of the RTS,S vaccine under a different formulation and with different participants. The results confirm the large effect size of RTS,S vaccination on MSP5 Abs and are shown in Supplemental Data 2. Additionally, vaccine-induced off-target reactivity to MSP5 in the first subset of qSAT (Luminex) measurements mainly involved increases in cytophilic IgG1 and IgG3 (Supplemental Data 3).

### Strong vaccination-induced off-target Ab levels decline but persist over 32 months of follow-up.

At M20, M21, and M32, off-target IgG levels with at least 2-fold increases at M3 (“strong” off-target Ab) remained significantly higher in the RTS,S group during follow-up time points despite a clear waning of Ab levels. Figure 1B shows their longitudinal trajectories, together with anti-CSP IgG levels for comparison. By contrast, most of the numerous Abs with small differences in levels (<2-fold) detected at M3 disappeared during follow-up or declined to an undetectable level given the sample size. Repeating the univariate screening for off-target Ab-level differences at follow-up time points, only 7 Ags were identified at M20, 11 Ags at M21, and 17 Ags at M32 with differences that passed the significance threshold, of which 5, 9, and 7 Ags, respectively, corresponded to the subgroup of strong off-target Ab at M3 (Supplemental Table 1 in Supplemental Data 1). The few Ab differences that had not been detected at M3 but were significantly different at later time points were always small in magnitude (<25%), mainly involved reductions in the RTS,S group, and included well-known markers of malaria exposure (e.g., MSPs, erythrocyte membrane proteins, etc).

### Booster vaccination reinforces vaccine off-target Ab increases.

The RTS,S/AS01E booster dose administered 18 months after primary vaccination (M20) increased the levels of strong off-target Abs. Overall, these Ab levels mimicked the characteristic waning and increasing pattern of anti-CSP Ab after primary and booster vaccination (Figure 1B). Similar to anti-CSP IgG, postbooster responses to these Ags did not reach levels as high as those following primary vaccination, a characteristic of RTS,S immunogenicity (5, 20). RTS,S/AS01E booster vaccination reinforced declining strong off-target Ab levels to at least 1.5-fold over comparators, with a majority above 2-fold.

Other malarial Ags also followed this pattern of increases detected following both primary and booster vaccination. In particular, 20 Abs were detected with significantly higher mean levels in the RTS,S-boosted group over comparators, and nearly all had also been increased following the primary dose (Supplemental Table 1 in Supplemental Data 1). The number of significant off-target Ab increases following the booster was smaller than after primary vaccination. This may be due to a combination of lower off-target Ab immunogenicity of the booster, as evident by smaller effect sizes of the induced increases, and to the decreased statistical power of the comparisons at M21 that used a smaller sample size.

### A single signature of vaccine off-target Ab responses correlated with anti- CSP Ab levels.

**Figure 2:**
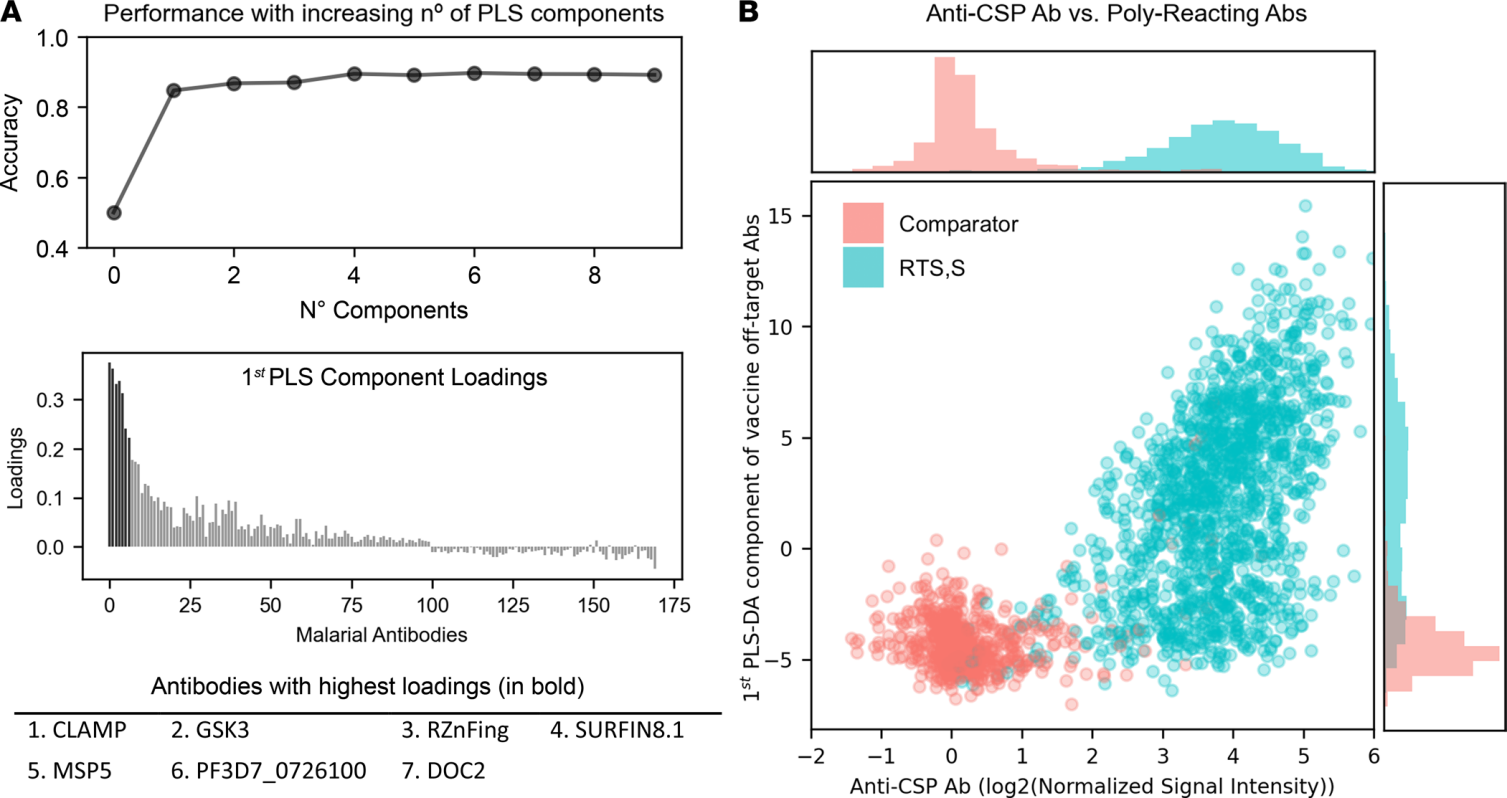
PLS-DA. All Ags with significant univariate differences (increases or decreases), but excluding CSP, were included as predictors in PLS-DA models with vaccination group as the outcome. (**A**) Top: cross-validated (5-fold) contribution to prediction accuracy of other components. Bottom: loadings of Ags to the first PLS-DA component. (**B**) Scatterplot of the first PLS-DA component scores against CSP Ab levels.

Off-target Ab responses to vaccination mainly occurred against multiple Ags simultaneously (cross-correlated) and displayed high levels of heterogeneity across RTS,S vaccinees. Using partial least squares discriminant analysis (PLS-DA) decomposition of all Ags presenting significant differences at M3, including increases and decreases, but excluding CSP from the panel, a single latent dimension of covariation with the vaccination group was identified. Figure 2A shows that beyond the first component, cross-validated performance scores did not improve and hit a ceiling of 80%–85% accuracy. Thus, vaccine-induced effects on off-target Ab levels did not happen independently. Vaccinees with off-target Abs strongly reacting to 1 Ag were very likely to also have Abs strongly reacting to other Ags in the signature.

When loadings were ranked by magnitude of fold differences between vaccination groups, the contribution of each Ag to the signature (PLS-DA first component loadings, Figure 2A, bottom) closely resembled the univariable differences in magnitude and sign; the same pattern of strong off-target Ab increases emerged, including CLAMP, SURFIN8.1, RZnFing, and so on, over a larger number of small contributions. Vaccine-induced off-target Ab changes, in addition to forming a single signature, also correlated with postvaccination anti-CSP Ab responses. Figure 2B shows anti-CSP Ab levels against the first PLS-DA component scores with a clear correlation within the RTS,S group (green dots, *P* = 0.48, CI [0.44–0.53]) but weak within the comparator group (red dots, *P* = 0.2, CI [0.12–0.26]). Thus, vaccinees with high anti-CSP Ab levels following RTS,S vaccination were more likely to present high off-target Ab increases.

Within a subset of the protein array samples for which Abs were measured by ELISA against the NANP repeat and C-terminal (C-Term) regions of CSP separately in the vaccinees, a similar correlation existed against only the NANP but not the C-Term region. Furthermore, high NANP region Ab avidity was also associated with higher off-target Ab responses, even after adjusting for NANP Ab levels (Supplemental Data 4). This association was stronger in cytophilic Ab subclasses (IgG1 and IgG3) compared with IgG2, IgG4, or IgM, which were also those most increased for anti-MSP5 Abs (Supplemental Data 3).

### Heterogeneity in the off-target Ab responses within RTS,S vaccinees.

**Figure 3:**
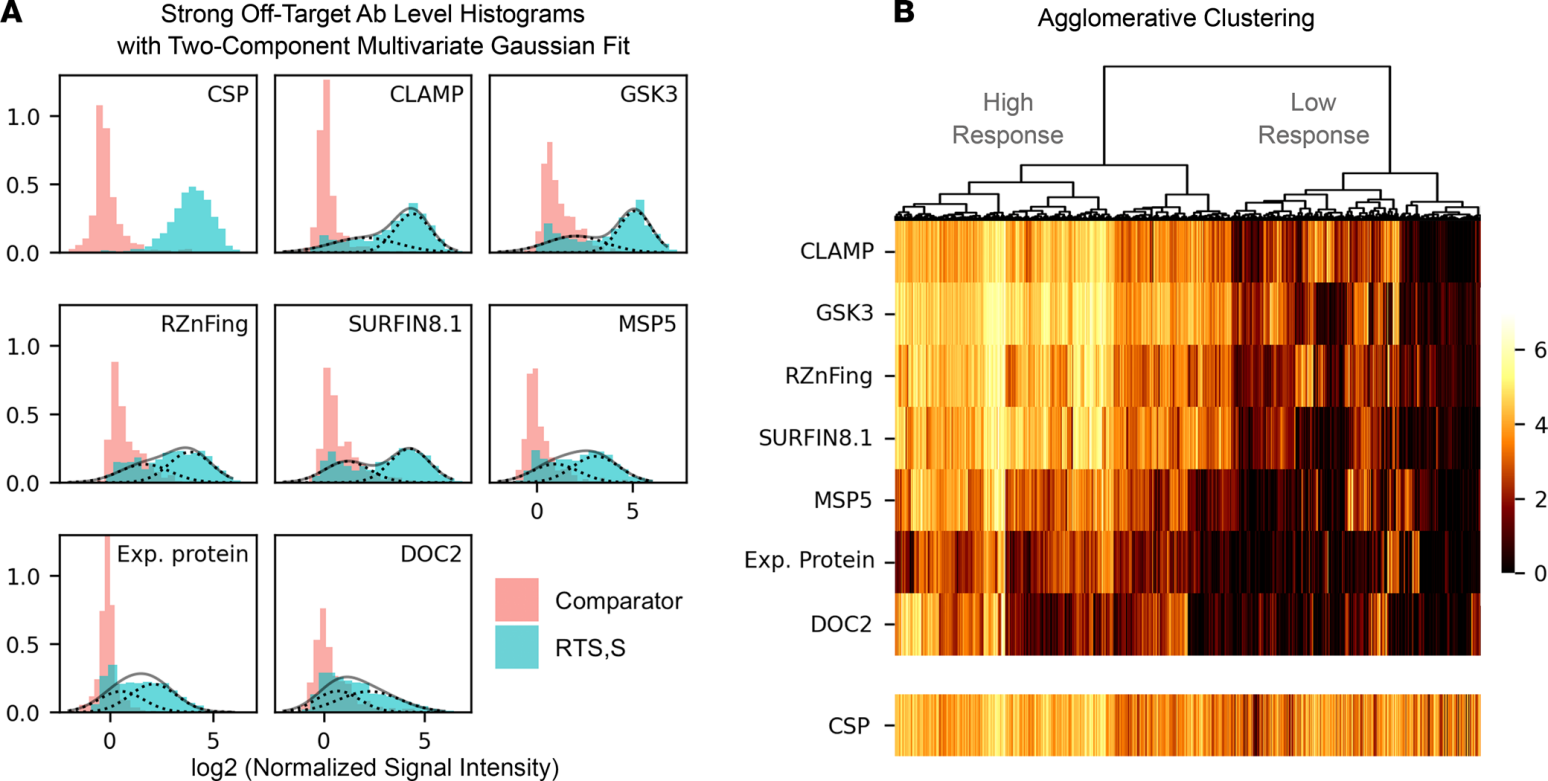
Bimodal heterogeneity in off-target Ab responses following RTS,S vaccination captured by 2 unsupervised classification algorithms. (**A**) The histograms show the log_2_-transformed normalized signal intensity for anti-CSP Abs and strong off-target Abs at M3. A multivariate 2-component Gaussian mixture distribution was fitted to the data, and the probability density curves for each component were overlaid (dashed lines are mixing Gaussian components; solid lines are the resulting mixture). (**B**) The heatmap of the log_2_-transformed normalized signal intensity for the strong off-target Abs (excluding anti-CSP) are shown where observations (vaccinees in the columns) are ordered according to an agglomerative clustering algorithm. The dendrogram on top depicts the order of clustering merging and informs about the dissimilarity between them as branch heights are proportional to cluster distances. Anti-CSP Ab levels are plotted as a reference.

Within RTS,S-vaccinated individuals, heterogeneity in off-target Ab responses was greater than that of anti-CSP. Figure 2B shows that the marginal histogram for off-target reactivity scores was flatter than that for anti-CSP levels. In Figure 3A, comparator vaccinees (red histograms) presented Ab levels mainly centered at 0 with relatively normal unimodal distributions, some slightly skewed rightward. This shows that comparators were mainly seronegative for anti-CSP Ab, as well as for strong off-target Abs. In contrast, RTS,S vaccinees presented Ab levels far above background. However, whereas anti-CSP levels were homogeneously increased and nearly all individuals were seropositive, off-target Abs presented a flatter distribution extending from 0 to very high levels, indicating a highly heterogeneous response to vaccination. Surprisingly, this heterogeneity appeared in the shape of bimodal distributions, with a first mode centered near 0 resembling that of comparators, and a second mode centered at high Ab levels, indicating strong seropositivity in only a subgroup of vaccinees. This characteristic was particularly notable for CLAMP, GSK3, RZnFing, and SURFIN8.1.

To explore if bimodality also occurred multidimensionally, i.e., seropositivity against a given off-target Ag was associated with seropositivity against other off-target Ags, a 2-component multivariate Gaussian mixture model was fitted. Figure 3A plots the predicted probability densities on top of the histograms to illustrate goodness of fit. The 2-component Gaussian mixture adequately captured the 2 subdistributions (putative underlying subgroups) in nearly all bimodal distributions. The algorithm classified 61% of all RTS,S-vaccinated individuals as high responders and provided a classification henceforth used to stratify vaccinated individuals. The binary classification was complemented with an alternative agglomerative clustering, a more agnostic algorithm that does not force the best classification to be binary. Nonetheless, Figure 3B shows that the main differences in off-target Ab responses to RTS,S vaccination were captured by the split of the top branch, confirming that a binary grouping was justified. Both classification algorithms, despite working differently, achieved 94% coincidence, a high score showing that the subgrouping of vaccinated individuals between low and high off-target Ab responders was robust.

### High off-target Ab responses are associated with age, malaria transmission intensity, and higher VE.

**Figure 4:**
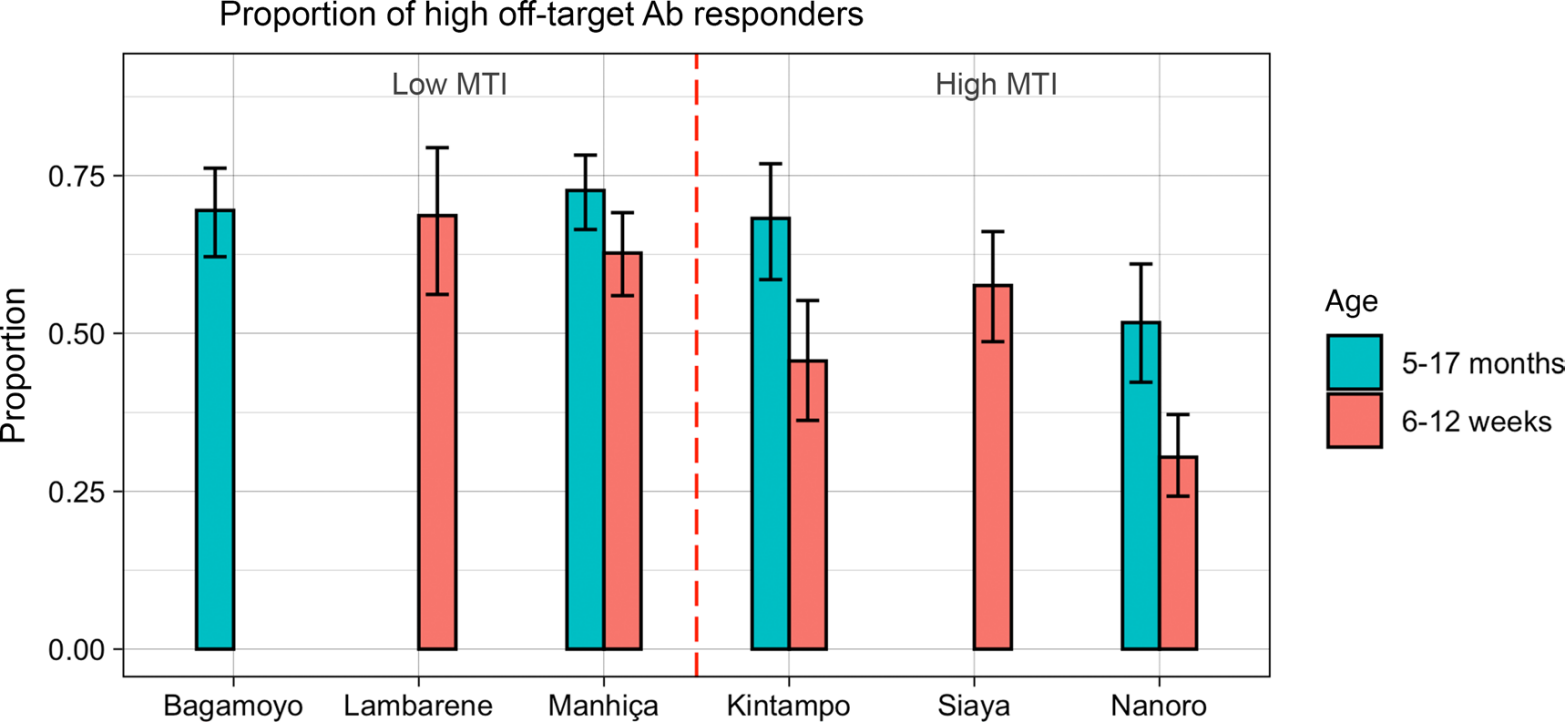
Association of vaccine off-target Ab reactivity with age group and MTI. Proportion of RTS,S vaccinees classified as high responders to vaccine off-target antigens is shown in the bar graph with 95% CIs for the proportions estimated as exact Clopper-Pearson binomial intervals. Missing bars are due to lack of data (i.e., samples not collected or selected) for the corresponding site.

Using the Gaussian mixture binary classification, we found that children are more often “high off-target Ab responders” than infants (Figure 4). In high malaria transmission intensity (MTI) sites, 59.6% of children (*n* = 184) were classified as high responders, and only 42.2% of infants (*n* = 293) were classified as high responders, a significant difference in proportions (*P* = 2.8 × 10^–5^). The same trend was observed in low MTI sites, with more high responders in children than in infants (71.3% [*n* = 134] and 64.1% [*n* = 191], respectively, *P* = 0.054). In addition, high off-target Ab responders were also more common in low than high MTI sites, as seen in the visual comparison by site and age of Figure 4. However, no differences were found in proportions of high off-target Ab responders between females and males (*P* = 0.99).

**Figure 5:**
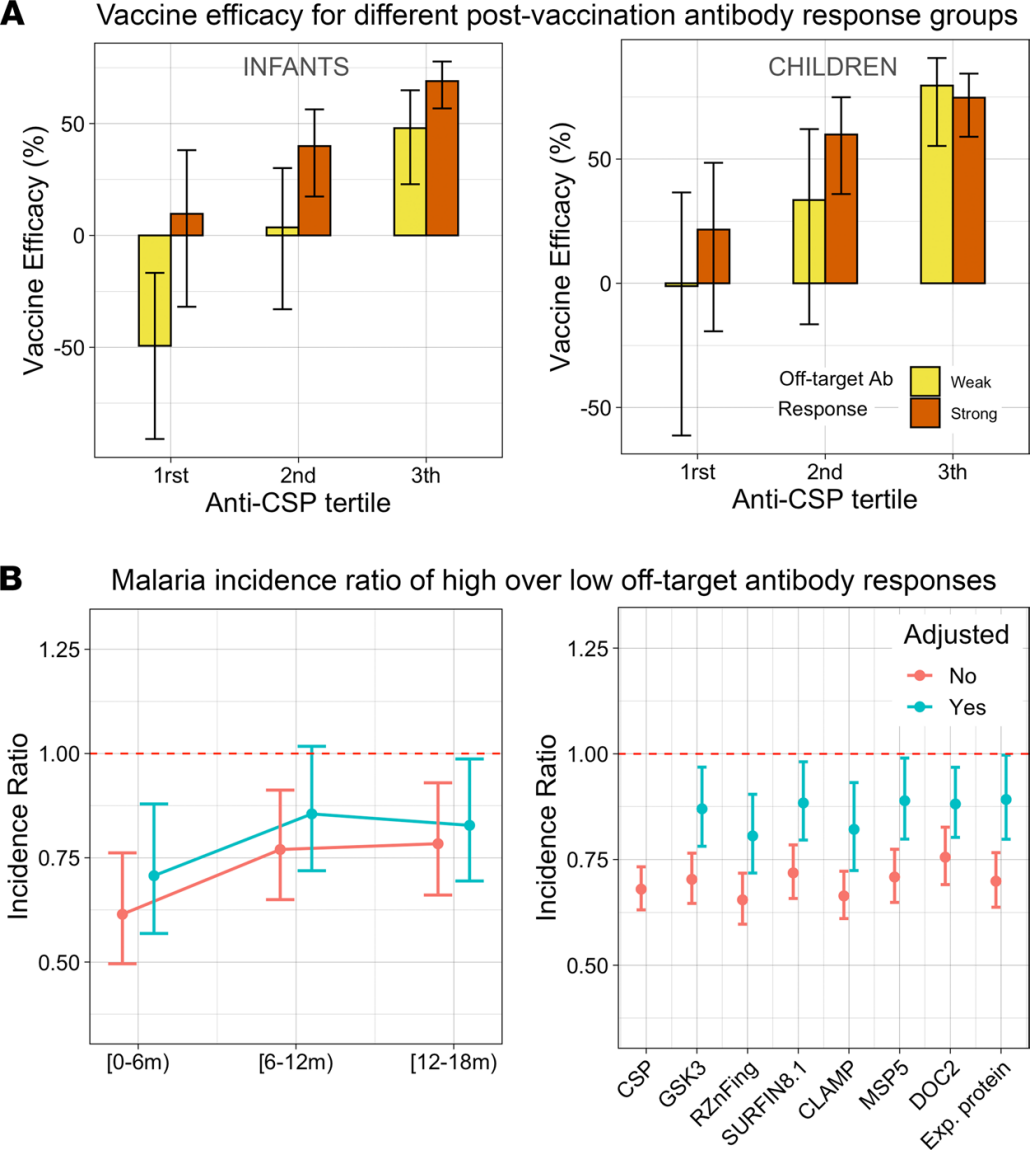
Association of the high off-target Ab responder group with VE. (**A**) VE over 1 year of follow-up since M3 was repeatedly calculated for 6 different RTS,S subgroups against the reference comparator group after estimating the corresponding incidence ratios (IRs) with negative binomial regression (VE = 1 – IR). Six postvaccination immunological response subgroups were defined based on anti-CSP levels (tertiles) and low versus high vaccine off-target Ab response according to our classification. (**B**) Left: clinical malaria incidence ratios were calculated within successive semesters after vaccination (M3) for high over low off-target responders. Right: incidence ratio increase per unit change of standard deviation of log_2_-transformed normalized signal intensity for vaccine off-target Abs with the largest increases. All plots contain estimates from models adjusted (red) and unadjusted by CSP levels (green). All models were adjusted by age group and site.

We calculated VE over 1 year of follow-up since M3 stratified by increasing anti-CSP levels (Ab level tertiles) and off-target Ab response (low vs. high responder classification). As expected, higher anti-CSP tertiles were associated with higher VE, in both infants and children (Figure 5). Despite large CIs in estimated VE as a consequence of low sample sizes in the RTS,S subgroups, RTS,S-vaccinated individuals classified as high off-target Ab responders tended to have higher VE than their low responder counterparts with similar anti-CSP levels.

The observed trend with VE could be driven by confounding variables, mainly site or residual anti-CSP differences in the subgroups. To adjust for age, site, and anti-CSP levels, malaria incidence was modeled including these factors as control variables, and incidence ratios between RTS,S vaccinees with high over low off-target Ab responses were estimated. When adjusting for age group and site, but not for anti-CSP, malaria incidence in high off-target Ab responders was significantly lower than in their low-responding counterparts for the 3 succeeding 6-month semesters after M3 (Figure 5B, left). The strongest difference in malaria incidence took place in the first semester, reaching an estimated 39% fewer cases (IR = 0.61, CI [0.50–0.76], *P* = 8.83 × 10^–6^) in high off-target Ab responders; in the second and third semesters, reductions became smaller (IR = 0.77, CI [0.57–0.91], *P* = 2.53 × 10^–3^; and IR = 0.78, CI [0.66–0.93], *P* = 5.20 × 10^–3^, respectively). When also adjusting for anti-CSP levels at M3 (including potential nonlinearities using b-spline basis functions), the association became weaker but clearly followed the same pattern, with a significant estimated malaria incidence reduction of 29% in the first semester (IR = 0.71, CI [0.57–0.88], *P* = 1.82 × 10^–3^) but smaller in the following semesters (second semester: IR = 0.86, CI [0.72–1.02], *P* = 0.08; third semester: IR = 0.83, CI [0.69–0.99], *P* = 0.034). This result indicates that part of the association of higher VE with vaccine off-target Ab reactivity cannot be explained by its correlation with anti-CSP levels alone.

To complement the previous analysis, instead of using the binary classification based on the joint distribution of off-target Ab responses, the association of malaria protection with strong off-target Ab levels was estimated (Figure 5B, right). When not adjusting for anti-CSP Ab levels, but still adjusting for age group and site, higher levels were significantly associated with protection to nearly the same degree as anti-CSP Ab levels. However, when adjusting for anti-CSP, their associations were weaker, with some no longer reaching statistical significance. No single off-target Ab clearly stood out as more predictive of protection than others.

### Sequence realignment between CSP and strongly reactive off-target Ags.

A BLAST of CSP including the NANP repeat and C-Term regions against the *P*. *falciparum* 3D7 strain ortholog of each strongly reacting off-target Ag identified no significant sequence similarity. We repeated the analysis using HBsAg coexpressed in the vaccine instead of CSP, again obtaining no significant results. A more guided BLAST of “NANP,” “NVDP,” or combination of “NANPNVDP” against full-length proteins also gave no significant matches, except for CLAMP. In the C-terminal proline-rich domain of CLAMP, there is a 5 aa NLNPN sequence (aa 381–385 of CLAMP) with a substitution of an alanine with leucine. To further explore short sequence alignments, we used a sliding 6 aa window (shifted 1 aa per window) of the NANPNVDP sequence with MAFFT on sliding 6 aa windows of the cross-reactive Ags. The proline in the CSP repeat region was considered an irreplaceable residue for providing structural rigidity in the epitope; therefore only 6 aa sequence windows containing at least 1 proline were included. The top hit for CLAMP was aa 381–386, containing the NLNPN sequence. Others had similar sequences with substitutions: for MSP5, the top sequence was an NSNPNL at aa 111–116; for RZnFing, it was NKNPNEN at aa 2072–2078; and PF3D7_0726100 had a sequence of 6 repeats each containing NNNPN flanked by tyrosine or aspartic acid residues. For DOC2, the top sequence aligned with the NVDP part of the CSP repeat was an NNDPN at aa 30–34, substituting a valine for an asparagine. GSK3 and Surfin8.1 had no clear linear sequence alignments with NANP or NVDP; however, it is possible that discontinuous sequences undetected by these methods could mimic the CSP repeat region. A limitation of this approach is our gap in knowledge of minimal epitopes for cross-reactive Abs. However, the physiochemical similarities in the top sequences of 5 of the strongly reacting off-target Ags suggests that a sequence of 5 to 6 aa may be sufficient and supports the postulate that the Ab binding to these proteins is cross-reacting IgG targeting the CSP.

## Discussion

We discovered considerable variation in Ab levels against 1000 malarial Ags shortly after vaccination in a large subset of children from the phase III clinical trial of the RTS,S/AS01E malaria vaccine. Significant differences involved a large number of probed Ags (17%), of which the majority were small in effect size. To our surprise, a small subgroup of these Ags presented highly increased Ab responses in the RTS,S vaccinees, nearly as high as anti-CSP Ab (4- to 8-fold increases over comparators). Furthermore, these vaccine-induced strong increases occurred only in one part of the group of vaccinated individuals, and strong off-target Ab responses were a predictor of increased protection, beyond what anti-CSP Ab levels alone could predict. Levels of strong off-target Ab only partially waned over follow-up months. At least 6 were still significantly increased nearly 3 years after primary vaccination (M32), even in the absence of a booster dose. As with anti-CSP IgG, the booster dose served to raise mostly the same strong off-target Abs and to delay their subsequent decline.

Given the interventional nature of the study, the cause of Ab level differences between groups can be attributed to RTS,S/AS01E vaccination. However, the causal effects of vaccination on off-target Ab profiles could be of 2 natures: A) directly and immediately induced by vaccination or B) indirectly and accumulating over time, due to the partial protection conferred by the vaccine, reducing exposure and thereby differentially acquired immunity. The temporal proximity of sampling after vaccination (M3 or M21) indicates that the majority of vaccine off-target Ab differences were directly induced by vaccination. In addition, at least 2 different biological mechanisms are possible for a direct effect of vaccination: 1) the large number of short-lived, small differences (<2-fold), including Ab increases and decreases, may result from a systemic perturbation of the immune system following vaccination, probably due to the immunostimulatory properties of the adjuvant, and 2) the subgroup of strong off-target Ab increases, comparable in magnitude to anti-CSP levels, may arise from epitope similarities between the RTS,S protein target and certain off-target Ags (i.e., cross-reactivity). Previous studies have shown that cross-reactivity between epitopes located in different malaria proteins is possible (16, 21). The fact that the intensity of the vaccine off-target Ab response followed a single signature and strongly correlated with postvaccination anti-CSP IgG levels, specifically with the NANP region, supports this hypothesis. Complementary assays measuring Ab subclasses for MSP5, one of the main off-target Ags and a well-known malaria vaccine candidate, showed that vaccine-induced off-target Ab increases involved mainly cytophilic IgG subclasses, the same that are increased in high off-target Ab responders for anti-NANP levels. Consequently, this observation provides further evidence that vaccine-induced Abs binding to MSP5 are cognate anti-CSP Abs, probably anti-NANP and mainly cytophilic IgG1 and IgG3, that may be cross-reacting. Sequence realignment analysis identified NANP-like stretches containing N and P repeats in some of these strongly reacting off-target Ags, in agreement with evidence that cross-reacting epitopes usually occur between immunodominant low-complexity repeat structures rich in asparagine and glutamate (17, 19). Their abundance in the malaria proteome is hypothesized to play a role in the evasion of the host’s immune response (18, 22).

Discarding the possibility of differential exposure indirectly influencing off-target Ab binding can be justified due to the assumption that reduced exposure in RTS,S vaccinees is expected to cause reduced antimalarial Abs, not increases. The hypothesis that increased subpatent parasite exposure due to partial protection afforded by RTS,S/AS01E vaccination would cause the increase in off-target Abs is also unlikely, in that sufficient exposure is unlikely to have occurred during primary vaccination or directly following the booster to elicit these responses, particularly in low MTI settings. Additionally, the off-target Ab responses decayed over follow-up, rather than accumulating. Off-target reactive Ags did not include typical markers of malaria exposure, such as apical merozoite antigen 1 or MSPs, with the exception of MSP5, which were decreased in RTS,S vaccinees as expected. Nearly all comparator vaccinees were seronegative for the strong reacting off-target Ags (their Ab levels were close to background). Taken together, the off- target Abs observed following RTS,S/AS01E immunization were most likely direct effects of vaccination.

Vaccine off-target reactivity also presents intriguing characteristics. Off-target Ab increases were cross-correlated, defining an “off-target Ab response signature” with heterogeneous responses for different vaccinated individuals. Because the intensity of this response also correlated with postvaccination anti-CSP levels, a parsimonious explanation could be that anti-CSP Abs and strongly reacting off-target Abs are the same, and the phenomenon would be a typical example of Ab cross-reactivity. However, this does not explain why there exists a substantial group of vaccinated individuals (around 40%) with very low vaccine off-target Ab levels, nearly as low as comparator vaccinees, while their anti-CSP Ab levels can be high or even very high. We captured this binary subgrouping of high versus low off-target Ab responders with multiple clustering techniques, although the pattern is most easily seen in the bimodal distributions of postvaccination off-target Ab levels in the RTS,S group. When a categorical grouping neatly emerges from data, it is often indicative of an underlying categorical determinant. However, we cannot rule out that bimodality may alternatively arise from a measurement limitation in the protein array technology. Yet, whether the heterogeneity in off-target Ab reactivity in vaccinated individuals is amenable to a binary subgrouping or, in reality, is not multimodal and simply spans a continuum, has few implications for the relevance of these results.

Vaccinated individuals presenting high off-target Ab responses had an estimated lower clinical malaria incidence. This association with protection was partially maintained as we adjusted for age, site, and postvaccination anti-CSP levels. In other words, vaccinated individuals with a high off-target Ab response to RTS,S/AS01E vaccination were more protected from infection than their vaccinated counterparts with similar age, site, and anti-CSP Ab levels. The result that high off-target Ab responses is associated with lower estimates of malaria incidence suggests that this immune signature may confer additional benefits, aside from being a proxy of high anti-CSP Ab levels, the best serological marker of VE thus far (11). One explanation is that vaccine off-target Abs binding to malarial Ags, possibly just anti-CSP IgG that are cross-reacting with them, are indeed playing a biological role in preventing symptomatic infections. CLAMP (in micronemes), SURFIN8.1 (in cell membrane), MSP5 (merozoite surface), DOC2 (host cell plasma membrane), and GSK3 (at Maurer’s cleft) are all cell surface proteins expressed at different stages of the *P*. *falciparum* parasite life cycle. Mounting an Ab-led immune response against them, assuming some must be sufficiently exposed at a certain stage of the parasite’s life cycle, could potentially be beneficial and reduce parasitemia. To further study this, it will be illuminating in the future to confirm specific functional characteristics of off-target Abs. Toward this, we have already shown that MSP5 Abs mainly encompass cytophilic IgG Abs and therefore those with the greatest ability to trigger effector functions related to complement fixation and opsonic phagocytosis.

However, our observations are also compatible with a scenario in which vaccine off-target Abs do not play any mechanistic role in protection and would simply be a proxy of the quality of the CSP response or other underlying protecting factors yet to be discovered. One such factor could be Ab maturation, since we found an association of high NANP Ab avidity with high Ab off-target responses after adjusting for anti-NANP Ab concentrations in a subset of samples with additional avidity measurements (Supplemental Data 4). Another factor could be a higher number of cytophilic anti-CSP IgGs as they were differentially increased in high off-target Ab responders.

The present study has 3 main implications for future research. First, it forewarns researchers investigating postvaccination naturally acquired immunity in populations vaccinated with RTS,S or other CSP-based vaccines of misinterpreting vaccine-induced off-target Ab changes. These studies usually focus on mid- to long-term immunological estimates and will likely encounter the long-lasting effects of vaccine-induced off-target Ab increases, which should not be mistakenly interpreted as markers of differential exposure in vaccinated populations. Second, having established that RTS,S-induced Abs bind not only to CSP but also to other unrelated malarial Ags, further research should investigate if these Ags are crucial to its life cycle and sufficiently exposed to immune effectors (e.g., surface expressed) for the off-target Abs to be beneficial to the human host. Third, further research is needed to determine whether off-target Ab reactivity is a common phenomenon also present after vaccination with other subunit vaccines and if it is indeed caused by cross-reactivity, as we suspect at least for the strong off-target Abs. If the latter were the case, then this discovery would have far-reaching implications beyond RTS,S/AS01E vaccination and malaria.

Cross-reactivity often occurs between strains of the same pathogen, close but different species, and even phylogenetically separated ones (heterologous or between-pathogen cross-reactivity) (15). Edward Jenner’s historical discovery of a vaccine against smallpox using inocula from cowpox was made possible, unknowingly, thanks to it. But, to our knowledge, no previous research has aimed to investigate the possibility of within-pathogen cross-reactivity, i.e., the capacity of a monoclonal Ab that has matured to bind to a cognate Ag from a given pathogen to additionally bind, perhaps with lower affinity, to other noncognate Ags from the same pathogen. Such a phenomenon may be common in nature (23, 24) but may have remained unnoticed due to lack of interest or technological limitations allowing broad Ag screening. However, the paradigm of the one-to-one lock-and-key Ag-Ab interaction is shifting toward a more nuanced several-to-several model. Some degree of cross-reactivity is finally acknowledged as 1) not uncommon if Abs are screened against a sufficiently large number of Ags and 2) potentially beneficial for the host and perhaps even favored by Ab clonal selection (25, 26).

An important limitation of this study is that protein microarrays do not necessarily detect conformational epitopes, and thus, the off-target Ab reactivity observed in this study may be to epitopes unexposed to Abs in native conformation. Another limitation is the possibility that some off-target reactive proteins were missed in the screen.

In this study, we have shown that high-throughput Ab screening in the course of vaccine trials can reveal broad vaccine-induced off-target Ab alterations, with some of these off-target Abs reaching impressive increases that are compatible with strong within-pathogen cross-reactivity. Importantly, the presence of strong off-target Abs was also a correlate of increased protection. Their corresponding Ags could potentially be candidates of multivalent next-generation RTS,S formulations as additive (or synergistic) responses with CSP.

## Methods

### Study design and data

This study was carried out in a subset of the phase III randomized clinical trial MAL055 (NCT00866619) and the MAL067 immunology study including infants (age 6–12 weeks at enrollment) and children (age 5–17 months at enrollment) from 6 African sites. The clinical trial tested safety, immunogenicity, and VE (4, 5). Participants received 3 doses of either the RTS,S/AS01E vaccine or a comparator vaccine (the meningococcal C conjugate in infants or rabies vaccines in children) at study months (M) 0, 1, and 2 for primary vaccination and a booster dose at M20. In the MAL067 study, blood samples were collected prior to the start of primary vaccination (M0), 3 months after (M3, 1 month after third dose), before the booster dose (M20), 1 month after it (M21), and at the end of follow-up (M32). Further details of sample size and demographic characteristics of the subsamples analyzed at each time point are reported in Table 1.

Sex, age group, and site were used as covariates either of interest or for adjustment purposes. Sites were classified as high or low MTI: Bagamoyo, Lambaréné, and Manhiça (representative of moderate/low MTI) had average annual malaria incidences in the non-RTS,S-vaccinated group after M3 of 0.2, 0.2, and 0.1, respectively; Kintampo, Nanoro, and Siaya (high MTI) had 2.3, 3.0, and 3.6, respectively. To model malaria incidence, we used the trial secondary clinical malaria case definition: illness in a child brought to a study facility with a measured temperature of 37.5°C or more, or reported fever within the past 24 hours, and *P*. *falciparum* asexual parasitemia at any density. No new episodes were registered during 14 days after an episode that met the case definition under evaluation to account for the short-term chemoprophylactic effect of antimalarial treatment. Furthermore, to correct for time at risk, the same 14 days were subtracted from the corresponding individual’s follow-up time.

### Antigen microarray data

The design of the protein microarray and procedures used in this study for IgG detection have been described elsewhere (27). Briefly, a partial proteome microarray with 1000 *P*. *falciparum* protein features (Pf1000, third generation) was developed at ADI. The 1000 full-length or partial *P*. *falciparum* proteins represent 762 genes from *P*. *falciparum* reference strain 3D7, including 61 PfEMP1s, vaccine candidate proteins, and 176 conserved *Plasmodium* proteins of unknown function.

Proteins were expressed using an in vitro transcription and translation (IVTT) system, the *Escherichia* *coli* cell-free Rapid Translation System kit (5 Prime). A library of partial or complete open reading frames (ORFs) subcloned into a T7 expression vector pXI has been established at ADI. This library was created through an in vivo recombination subcloning process with PCR-amplified ORFs, and a complementary linearized expressed vector transformed into chemically competent *E*. *coli* was amplified by PCR and subcloned into pXI vector using a high-throughput PCR recombination subcloning method described elsewhere (28). Each expressed protein includes a 5′ polyhistidine (HIS) epitope and 3′ hemagglutinin (HA) epitope. After expressing the proteins according to manufacturer instructions, translated proteins were printed onto nitrocellulose-coated glass AVID slides (Grace Bio-Labs, Inc.) using an Omni Grid Accent robotic microarray printer (Digilabs, Inc.). Each slide contained 8 nitrocellulose “pads” for which the full array was printed in replicate, allowing 8 samples to be probed per slide. Microarray chip printing and protein expression were quality checked by probing random slides with anti-HIS and anti-HA monoclonal Abs with fluorescence labeling.

Prior to sample application, a probing plan was developed to balance serum/plasma samples across microarray slides by the following variables: sample collection time point, clinical trial site, age group, sex, and case-control status during the first 12 months of follow-up postvaccination. Paired samples from a single trial participant were probed onto a single chip (8 samples per chip, up to 5 paired samples per chip) or subsequently printed chips to minimize variation for time point comparisons.

Samples were probed on the Pf1000 microarrays at 1:100 with a 3 mg/mL DH5α *E*. *coli* lysate (GenScript, custom preparation) overnight at 4°C. Secondary anti-IgG Ab (polyclonal biotin-SP-conjugated donkey anti–human IgG; Jackson ImmunoResearch, catalog 709-065-098) was applied the following day at 1:1000 (respectively in 2% *E*. *coli* lysate; GenScript) for 1 hour at room temperature. Streptavidin Sensilight-P6824 (Columbia Biosciences, catalog D7-2212) was applied to each slide at 10 μg/mL (2% *E*. *coli* lysate; GenScript) for 1 hour, covered (no light) at room temperature. Washes were performed with Tween Tris-buffered saline 3 times before/after all incubations. Prior to overnight drying in a desiccator, all slides were washed with TBS (Tris-buffered saline) 3 times and spun-dry by centrifugation at 1000*g* for 4 minutes. Air-dried chips were scanned on a GenePix 4300A High-Resolution Microarray Scanner (Molecular Devices), and spot and background intensities were measured using an annotated grid file (.GAL). Data were exported in Microsoft Excel.

Raw spot and local background fluorescence intensities, spot annotations, and sample phenotypes were imported and merged in the R statistical environment, where all subsequent procedures were performed. Foreground spot intensities were adjusted by local background by subtraction, and negative values were converted to 1. The data set was normalized to remove systematic effects by dividing by the median signal intensity of the IVTT controls for each sample. Since the IVTT control spots carry the chip, and sample and batch-level systematic effects, but also Ab background reactivity to the IVTT system, this procedure normalizes the data and provides a measure of the specific Ab binding relative to the nonspecific Ab binding to the IVTT controls (a.k.a. background). Thus, the normalized signal intensity is the intensity signal-to-noise ratio. In the manuscript, we often make use of the log_2_-transformed normalized signal intensity, whereby a value of 0 represents Ab levels equivalent to the background, a value of 1 represents Ab levels twice that of the background, and each subsequent unit increase represents a doubling of Ab levels.

### Data characteristics

RTS,S and comparator vaccinees in the study subsample were similar with regard to pretreatment demographic and epidemiological characteristics such as age group, sex, and site (Table 1). In particular, imbalances were small (<1%) for age group, but slightly greater for site and sex (<5%) with regard to all time points, except for M32 when a considerable difference in proportion of sites existed (15%). To prevent potential biases, all analyses comparing vaccination groups were adjusted for these variables. Malaria incidence was calculated using all participants who had M3 samples. Follow-up intervals always started at M3 (blood sampling, performed approximately 1 month following the end of primary vaccination) and ended 6, 12, or 18 months afterward depending on analysis. Participants who were lost to follow-up were less than 5% in either vaccination group (Table 1). The analysis involving malaria protection or VE was restricted to participants who were not lost to follow-up (complete case analysis). However, the analysis was repeated including individuals who were lost to follow-up, after adjusting for their shorter time at risk in the offset of the negative binomial, to show robustness against the small number of dropouts (Supplemental Data 5).

### Statistics

#### Univariate Ab differences between vaccination group.

For each time point and Ab response to the microarray Ags, a linear model was fit with vaccination group as the variable of interest and age group, site, and sex as control variables to account for their slight imbalances between vaccination groups (Table 1). The outcome variable was the log_2_-transformed normalized signal intensity of each probed Ag in the microarray. Therefore, the estimated linear coefficient encoding the vaccination group difference, when exponentiated, could be interpreted as point estimates of geometrical mean fold differences in Ab levels of one vaccination group over the other, holding the control variables fixed. Regression coefficient standard deviations were used to generate 95% CI. *P* values were obtained from 2-tailed *t* tests. Only differences that were significant at a false discovery rate of 5% following the Benjamini-Hochberg method were reported (29).

#### PLS-DA to capture postvaccination Ab latent potential signatures.

A PLS decomposition of all microarray responses to malarial Ags was conducted to find latent signatures that maximally correlated with the vaccination group. Anti-CSP Ab levels were removed from the panel to explore covariation with levels beyond the RTS,S target Ag. The PLS1 algorithm was used with 500 maximum number of iterations of the NIPALS inner loop as is implemented by Scikit-learn (version 0.20). To study the number of relevant latent dimensions of covariation with the vaccination group, 5-fold cross-validation was used to predict the accuracy of the PLS-DA models with an increasing number of components.

#### Unsupervised classification of vaccinated individuals according to their postvaccination Ab profiles.

Two different unsupervised algorithms were used to classify RTS,S/AS01E vaccinees into 2 subgroups according to their increase in off-target Ab levels following vaccination, again excluding anti-CSP from the panel.

For multivariate 2-component Gaussian mixture, due to the bimodal distributions observed in the log-transformed Ab normalized signal intensities, a 2-component multivariate Gaussian fit was used, with estimated full covariance (no constraints) for each component, to model an empirical probabilistic distribution based on 2 underlying classes with different mean, covariance, and assumed normal distribution. This empirical distribution was used to classify observations based on their probability of belonging to one of 2 subgroups of vaccinees, as well as to classify unseen new data.

For hierarchical agglomerative clustering, the same analysis was repeated using a more agnostic classification algorithm that does not require an a priori choice of the number of resulting classes: hierarchical clustering that iteratively merged data observations pairwise, i.e., RTS,S vaccinees according to their postvaccination off-target Ab levels, into increasingly large clusters, ordering the merges using the Ward variance minimization algorithm (30). Successive partitions or clusters were obtained, hierarchically ordered from small to large and outputted as a measure of distance between merging clusters at each level. Visual representations such as dendrograms and sorted heatmaps were used to explore multivariate relationships, not necessarily linear, and visualize the emergence of types and subtypes.

For both unsupervised classification algorithms, their Scikit-Learn implementation was used (31).

#### Association of clinical malaria incidence with vaccine off-target Ab signatures.

Clinical malaria incidence (number of infections and reinfections over a follow-up time) was modeled with negative binomial regression, a probabilistic model where the number of infections and reinfections is assumed to follow a negative binomial distribution with the logarithm of the average incidence conditional on a weighted sum of predictors. The logarithm of the follow-up time was added as an offset variable to account for different times at risk between individuals. Having used a log link function, the exponentiated regression coefficients automatically become IRs of a given level in a categorical predictor over the reference level. For continuous predictors, estimated IRs refer to the comparison of malaria incidence within 2 populations with a unit change difference in the continuous predictor. Whenever IR was estimated for continuous variables, the predictor was standardized, i.e., unit changes became unit standard deviation increases (or decreases). Negative binomial regression was also used to estimate VE for different subgroups of RTS,S vaccinees classified according to their vaccine off-target Ab levels with the formula VE = 1 – IR, where IR is specifically the ratio of malaria incidence within the RTS,S subgroup of interest over that of the comparator group. Negative binomial regression coefficient standard deviations were used to generate 95% CI and *P* values were obtained from 2-tailed Wald’s tests on the corresponding coefficients. *P* < 0.05 was considered statistically significant. The glm.nb function from the MASS R package (32) was used to fit negative binomial regressions.

### Study approval

The multicenter study protocol was approved by the Comitè Ètic d’Investigació Clínica (Hospital Clínic Research Ethics Committee, ref 2008/4622), Barcelona, Spain, and the Research Ethics Committee for PATH, Seattle, Washington, USA. Written informed consent was obtained from parents or guardians before the start of the work.

## Author contributions

C Dobaño, GM, JJC, C Daubenberger, BM, BG, SK, STA, HT, SOA, and KPA designed research studies; JJC, CJ, AJN, MM, H Sorgho, DD, OT, GM, C Dobaño, and C Daubenberger conducted experiments; JJC, CJ, AJN, MM, H Sorgho, DD, OT, C Dobaño, GM, C Daubenberger, BM, BG, SK, STA, HT, KAK, KPA, and SOA acquired data; C Dobaño, GM, JJC, and NAW coordinated studies; DM, JJC, AR, AO, H Sanz, CV, and PP analyzed data; DM, JJC, GM, C Dobaño, and PP wrote the first draft of the manuscript; and all critically reviewed the manuscript and approved the final version. The method used in assigning the authorship order among co–first authors was considering contributions to the final data analysis reported here and manuscript write-up.

## Supplementary Material

Supplemental data

Supplemental table 1

## Figures and Tables

**Table 1:**
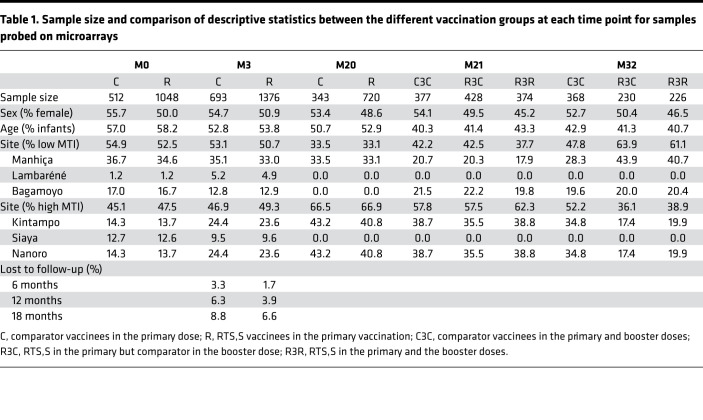
Sample size and comparison of descriptive statistics between the different vaccination groups at each time point for samples probed on microarrays

## Version 1 04/21/2022

In-Press Preview

## Version 2 05/23/2022

Electronic publication
