# Supplementary material for: Strong off-target antibody reactivity to malarial antigens induced by RTS,S/AS01E vaccination is associated with protection
Source: JCI Insight. 2022 May 23;7(10):e158030. doi: 10.1172/jci.insight.158030 (PMC9220828; doi:10.1172/jci.insight.158030)
Supplement: Supplemental data [file jciinsight-7-158030-s145.pdf]

Supplementary Material 2. Merozoite Surface Protein 5 (MSP5) antibody (Ab) increases following RTS,S vaccination measured with different immunoassays and in another clinical trial

1. Presentation of replication datasets

We investigated MSP5 Ab increases one month following RTS,S primary vaccination in two other datasets for which we also had MSP5 Ab measurements:

1) MSP5 IgG responses from 195 young children and infants from a nested immunological study of the phase 3 clinical trial of the RTS,S/AS01E vaccine (MAL055 NCT00866619) using quantitative suspension array technology (qSAT) (1).

2) MSP5 IgGs responses from 813 children age 1-4 yrs old from a nested immunological study of a phase 2b clinical trial of the RTS,S/AS02 vaccine using qSAT (Jairoce C, in preparation)

Dataset 1) is a subsample of the same phase 3 vaccine trial from which the microarray samples were obtained in the main manuscript. The overlap between the two studies is only partial: out of 195 qSAT samples, 139 children were also included in the microarray substudy. Therefore dataset 1) can be regarded as a replication in a subset of nearly the same data using a different measurement technology.

By contrast, 2) is a completely independent dataset, with different participants, of a different age range, and a different formulation of the RTS,S vaccine in terms of the adjuvant.

Other off-target antigens that were found increased in the microarray were not included in the panels from the above studies and could not be replicated.

2. Three MSP5 protein constructs probed with the antigen microarray

In the 1000 *P. falciparum* microarray used for the manuscript, we assayed three different constructs from the full MSP5 protein. Below their a.a. sequences:

MSP5 (i), PF3D7_0206900.1.1o2 :
MNILCILSYIYFFVIFYSLNLNNKNENFLVVRRLMNDEKGEGGFTSKNKENGNNNRN
NENELKEEGSLPTKMNEKNSNSSDKQPNDISHDESKSNSNNSQNIQKEPEEKENSNPN
LDSSENSSESATRSVDISEHNSNNPETKEENGEEPLDLEINENAEIGQEPPNRLHF

MSP5 (ii), PF3D7_0206900.1.e1 :
MNILCILSYIYFFVIFYSLNLNNKNENFLVVRRLMNDEKGEGGFTSKNKENGNNNRN
NENELKEEGSLPTKMNEKNSNSSDKQPNDISHDESKSNSNNSQNIQKEPEEKENSNPN
LDSSENSSESATRSVDISEHNSNNPETKEENGEEPLDLEINENAEIGQEPPNRLHFD

MSP5 (iii), PF3D7_0206900.1.2o2:
NVDDEVPHYSALRYNKVEKNVTDEMLLYNMMSDQNRKSCAINNGGCSDDQICININ
NIGVKCICKDGYLLGTKCIILNSYSCHPFFSILIYITLFLLLFV

As reported in the manuscript and ***Supplementary File 1***, MSP5 (i) was strongly increased following vaccination (M3), 4.0, 95% CI [3.7, 4.4]. MSP5 (ii) was also increased but less (1.99 CI [1.87, 2.13]). Finally, MSP5 (iii) was not increased significantly (1.01 CI [0.98, 1.04]).

In the replication datasets, we measured IgG responses using another MSP5 construct, that is the same for both studies but different from those in the microarray. Below we reference its a.a. sequence, highlighting in yellow the overlap with the microarray MSP5(i), in red with MSP5(ii) and underscored with MSP(iii):

MNILCILSYIYFFVIFYSLNLNNKNENFLVVRRLMNDEKGEGGFTSKNKENGNNNRN
NENELKEEGSLPTKMNEKNSNSSDKQPNDISHDESKSNSNNSQNIQKEPEEKENSNPN
LDSSENSSESATRSVDISEHNSNNPETKEENGEEPLDLEINENAEIGQEPPNRLHFDNV
DDEVPHYSALRYNKVEKNVTDEMLLYNMMSDQNRKSCAINNGGCSDDQICININNI
GVKCICKDGYLLGTKCIILNSYSCHPFFSILIYITLFLLLFV

The difference between MSP5 (i) and MSP5 (ii) of a single additional aspartic acid ("D") is notable because the additional amino acid is separated from the prior string by an intron in the current annotation on PlasmoDB.org. Thus, since genomic DNA was used for cloning, lower cloning efficiency in MSP5 (ii) may account for the difference in signal strength.

3. MSP5 IgG increases from a nested immunological study of the same phase 3 RTS,S/AS01E vaccine trial using qSAT

We measured IgG responses, assayed using qSAT applying the xMAP™ technology (Luminex Corp., Texas), from 195 young children vaccinated with either RTS,S/AS01E (n=129) or a comparator vaccine (n=66), one month after the last dose of the primary vaccination (M3). Further details on sample characteristics and data acquisition can be obtained from published results (Dobaño et al., 2019).

For this study, we estimated a four times larger geometric mean of MSP5 Ab levels of RTS,S over comparator vaccinees one month following primary (fold-increase of 4.0, 95% CI [2.4, 6.6]). Of note, the point estimate fold increase is here very similar to the one we estimated for the protein construct MSP5 (i) from the antigen microarray.

Out of 195 qSAT samples, 139 children were also included in the microarray substudy, allowing us to pair both measurements and investigate their agreement. Figure 1 shows that the greatest correlation of the qSAT MSP5 measurements is with the MSP5 (i) protein construct of the microarray. This result supports that the putative cross-reacting epitope should lie in their overlapping sequences (highlighted in yellow).

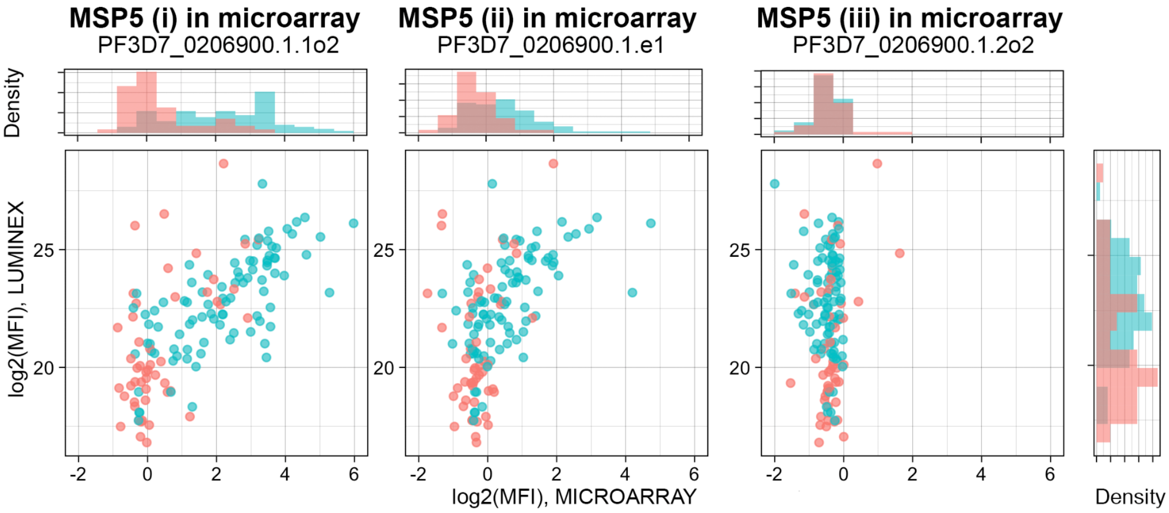

Figure 1. MSP5 Ab levels probed with qSAT (y-axis) against three different constructs of MSP5 probed with a protein array (x-axis). *X-axis values are different for each of the three panels (three different protein constructs) but y-axis values do not and produce a single marginal histogram (on the right). Red corresponds to comparator, green to RTS,S vaccinees.*

We also see that the marginal plots from Figure 1 do not show a clear bimodality in RTS,S MSP5 Ab levels. This could be due to insufficient sample size, given that we cannot clearly see it in the microarray subset either. On a different note, we also learn that the microarray measurements may not be able to resolve differences at low Ab levels where measurements are packed around zero (background MFI), whereas differences at this low dynamic range can be better resolved with qSAT. The fact that the protein array may poorly resolve low Ab levels and only start measuring at a certain Ab concentrations could also explain why a potential continuous heterogeneity in Ab responses (approximately log-normal in normalized MFI) takes the shape of a bimodal distribution, the first mode being the result of measurement left-censoring at low concentrations, the second mode being the real Ab level order of magnitude peak.

4. MSP5 IgGs responses from 813 children from a nested immunological study of a separate phase 2b clinical trial

We measured MSP5 IgG responses from 813 children (age = 12-60 months) vaccinated with either RTS,S/AS02A (n=124) or a comparator vaccine (n= 696) (1).

Again, we estimated the fold increase in the geometric mean of MSP5 Ab MFI levels of RTS,S over comparator vaccinees one month following primary vaccination (2.2 fold-increase, 95% CI [1.8, 2.8], CI obtained with bootstrap N=2000). This estimated fold-increase is lower than those obtained from the protein microarray and qSAT. Disagreement may have several sources since age range, vaccine preparation and MSP5 constructs are different. Furthermore, in Figure 2 $\log_{10}$ MFI measurements are saturated (at about >4.25). This saturation (right censoring) involves underestimating the true expected differences in Ab level means, as a greater number of RTS,S participants with high MSP5 Ab levels had their Ab levels under-measured than comparators. Trajectories in Figure 2 also show that MSP5 Ab that were increased one month after primary vaccination in the RTS,S group later attenuate and are not found 8.5 months after.

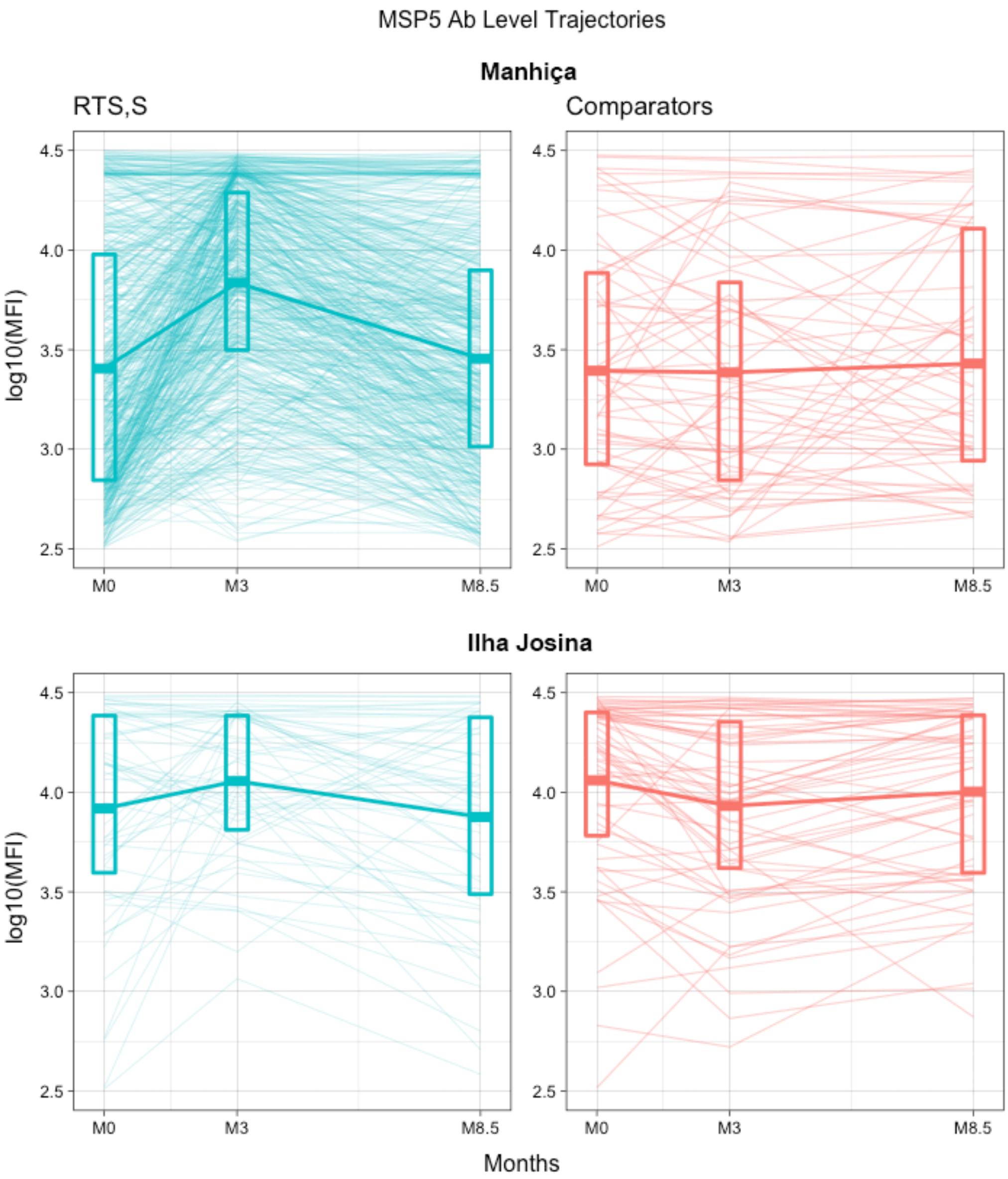

Figure 2. MSP5 Ab level trajectories before primary vaccination (M0), one month after it (M3) and 8.5 months at follow-up (M8.5) for different vaccination groups. *Thin lines on the background correspond to individual trajectories. Crossbars correspond to group summary statistics per time point with 1st, mean and 3rd quartiles represented. TOP: Manhiça cohort representative of low malaria transmission intensity; BOTTOM: Ilha Josina cohort representative of high malaria transmission.*

References

1. Dobaño C, Ubillos I, Jairoce C, et al. RTS,S/AS01E immunization increases antibody responses to vaccine-unrelated Plasmodium falciparum antigens associated with protection against clinical malaria in African children: a case-control study. BMC Med. 2019;17(1):157. Published 2019 Aug 14. doi:10.1186/s12916-019-1378-6

2. Alonso PL, Sacarlal J, Aponte JJ, et al. Efficacy of the RTS,S/AS02A vaccine against Plasmodium falciparum infection and disease in young African children: randomised controlled trial. Lancet. 2004;364(9443):1411-1420. doi:10.1016/S0140-6736(04)17223-1

Supplementary Material 3. Vaccine off-target MSP5 reactivity mainly occurs in cytophilic IgG1 and IgG3 subclasses

We measured IgG1, IgG2, IgG3, IgG4 and IgM levels against MSP5 from 195 young children and infants (170 overlapping with the protein array) using quantitative suspension array technology (qSAT) antibody (Ab) measurements (same experiment as referred in Supplementary 2, with acquisition and pre-processing details in previous published studies (1, 2)). Furthermore, we repeated the same analyses for anti-CSP IgG subclasses, keeping in mind our hypothesis that some of the RTS,S-induced anti-CSP Abs may be cross-reacting against off-target antigens such as MSP5.

Following primary vaccination (M3), IgG subclass levels to MSP5 in the different vaccination groups showed the following pattern: 1) no significant differences between comparator vaccinees and RTS,S in low off-target Ab responders, in agreement with one of the main results obtained from the protein array (i.e. a large group of RTS,S vaccinees had a post-vaccination off-target response similar to comparator vaccinees), 2) RTS,S high off-target Ab responders had increased Ab levels over comparators and also over low responders. However, a differential pattern was seen depending on IgG subclass: large increases in IgG1 (17.8 fold increase in MTI geometric means of high over low off-target RTS,S responders, 95% CI [8.89, 35.68], p=1.5e-13) and IgG3 (6.03 fold increase, CI [3.49, 10.41], p=1.48e-09); moderate in IgG2 (3.62, CI [2.10, 6.29], p=8.49e-06), and nearly no increases in IgG4 (1.39, CI [1.12, 1.71], p=0.02) and in IgM (1.65, CI [1.05, 2.59], p=0.031) (Supplementary Figure 1).

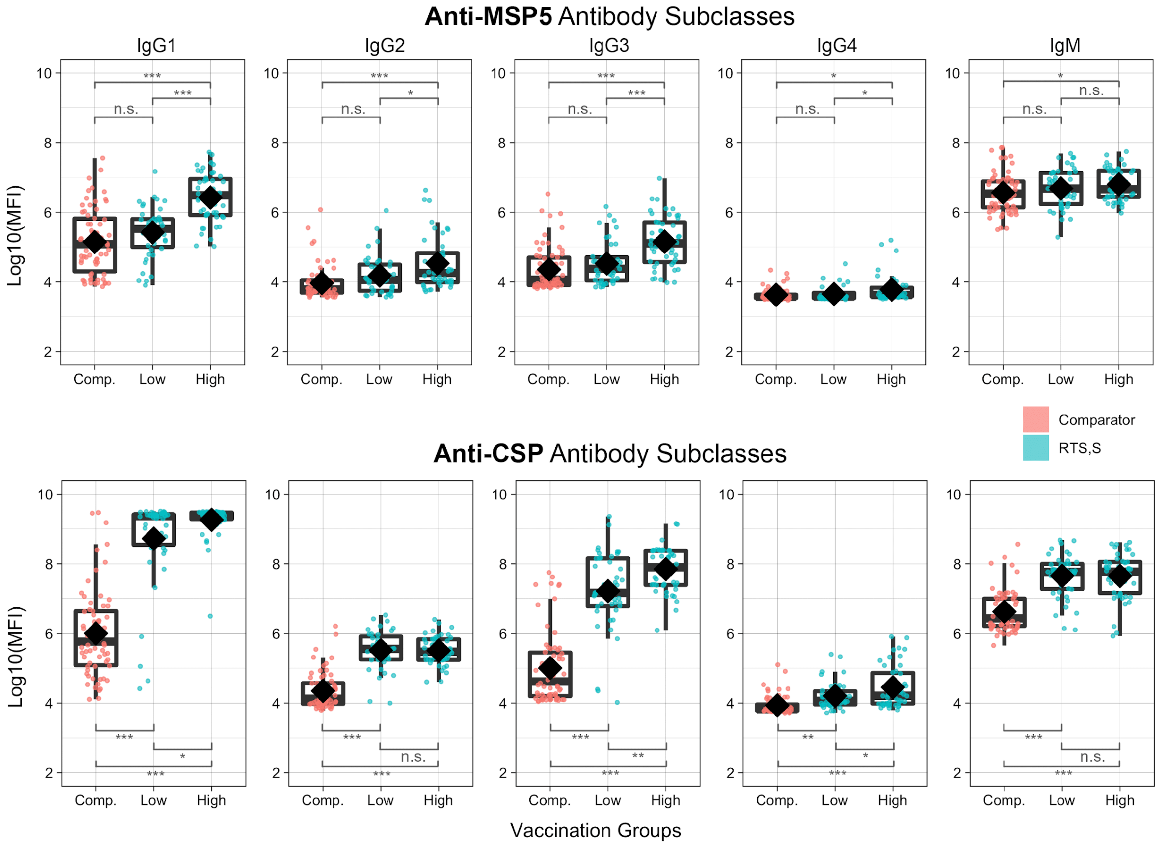

Figure 1. Differences of anti-MSP5 and anti-CSP subclass IgG levels between Comparators, RTS,S Low and RTS,S High off-target antibody responders*. Boxplots*

*illustrate the medians and the 25th and 75th quartiles, diamonds show the geometric mean, whiskers display the 1.5 interquartile ranges, and coloured dots are measured data. Significance of the geometric mean differences are calculated as t-tests on the regression coefficients in models adjusted by site and age group (n.s., *, **, and *** stand for p-values in the following ranges >0.05>0.005> , respectively).*

Regarding anti-CSP IgG subclasses, as reported previously (1), RTS,S vaccination induces very strong responses mainly to the cytophilic subclasses IgG1 and IgG3, smaller in IgG2 and IgM, and nearly undetectable in IgG4. Here the novelty lies in stratifying RTS,S vaccinees into high vs. low responders based on their off-target Ab response. RTS,S primary vaccination induced higher IgG1 levels in high off-target responders (3.66 fold increase over low responders, CI [1.27, 10.56], p=0.017), also higher in IgG3 (4.23, CI [1.66, 10.77], p=0.003), only moderately higher in IgG4 (1.717, CI [1.14, 2.57], p=0.01) but no significant differences in IgG2 and IgM.

Data show that those anti-MSP5 IgG subclasses that are mostly increased in high off-target Ab responders, are also those mostly increased for anti-CSP levels. This observation provides further evidence that vaccine-induced Abs binding to MSP5 are probably cognate anti-CSP Abs, mainly cytophilic IgG1 and IgG3, that may be cross-reacting.

The fact that vaccine-increased off-target Abs (anti-MSP5 at least) are mainly IgG1 and IgG3 also has implications for their potential role in increased protection. IgG1 and IgG3, are the most effective subclasses in the induction of effector functions such as complement fixation and Fc receptor binding, thus being capable, not only of potentially neutralising an invading parasite, but also of leading a more complex antiparasitic Ab-mediated effector response in cooperation with innate immune cells. On the other hand, IgG3 has the shortest half-life and this should have implications in the duration of increased protection in high off-target Ab responders that, according to our study, rapidly waned and nearly disappeared six months following the primary vaccination. Figure 2 shows the time trajectories between vaccination groups for anti-MSP5 and anti-CSP Abs. The trajectories clearly show that anti-CSP IgG3 declines faster than the other subclasses. However, it is more difficult to see such differential decline for MSP5 Ab subclasses because the vaccine-induced increases are of a smaller effect size.

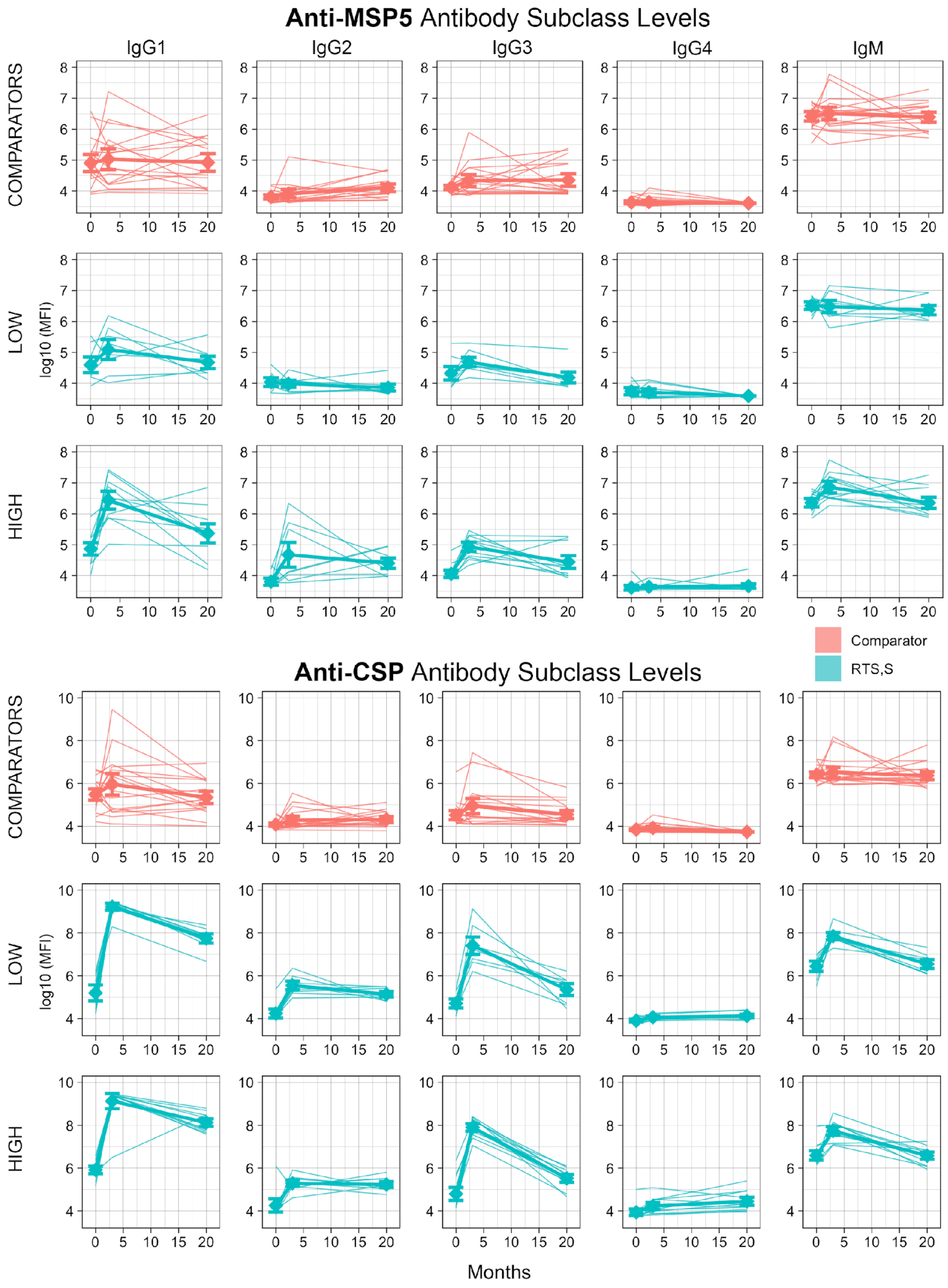

Figure 2. Longitudinal trajectories of IgG subclass levels against MSP5 and CSP antigens. *Thin lines correspond to individual repeated measurements (M0, M3 and M20); thick lines correspond to geometric means with whiskers indicating 95% for comparator vaccinees (17 participants in red, top row) and for RTS,S vaccinees (green), separating between low (8 participants, middle row) and high (11 participants, bottom row) antibody off-target responders.*

References

1. Ubillos I, Ayestaran A, Nhabomba AJ, et al. Baseline exposure, antibody subclass, and hepatitis B response differentially affect malaria protective immunity following RTS,S/AS01E vaccination in African children. BMC Med. 2018;16(1):197. Published 2018 Oct 31. doi:10.1186/s12916-018-1186-4.

2. Dobaño C, Santano R, Vidal M, et al. Differential Patterns of IgG Subclass Responses to Plasmodium falciparum Antigens in Relation to Malaria Protection and RTS,S Vaccination. Front Immunol. 2019;10:439. Published 2019 Mar 15. doi:10.3389/fimmu.2019.00439.

Supplementary Material 4. Association of vaccine off-target IgG response with NANP repeat and C-terminus CSP IgG concentration and avidity measured by ELISA

To further study the specificity and strength of association between post-vaccination anti-CSP and vaccine off-target IgG levels at M3, antibody (Ab) responses from a subsample of 777 participants (536 RTS,S/AS01E vaccinees) for whom we had ELISA IgG concentration and avidity data against the NANP repeat and C-terminal (C-Term) regions of CSP, were compared to protein array measurements. Details of the ELISA assays and data processing were reported previously (Dobaño et al., 2019).

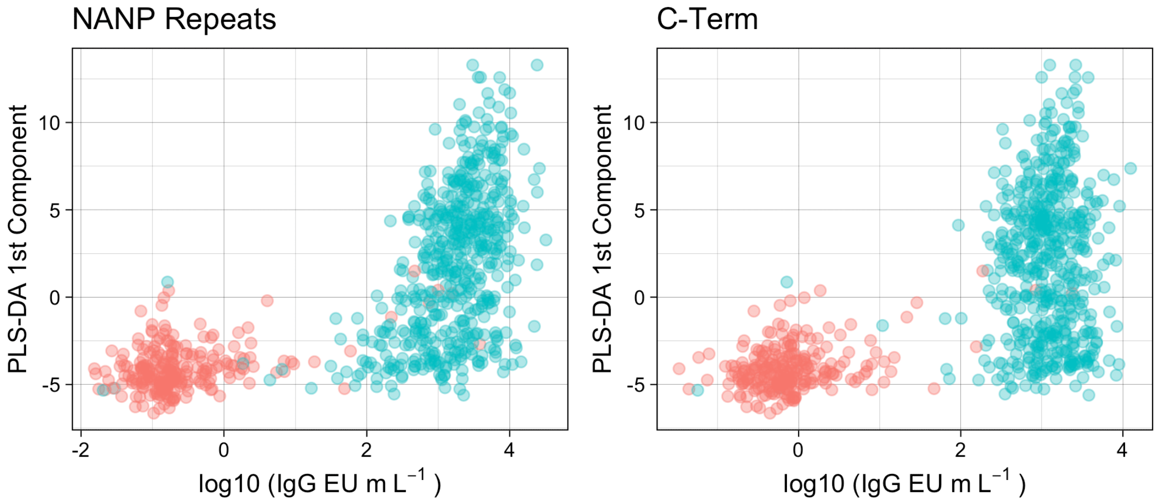

Figure 1. Association of NANP repeat and C-Term CSP IgG concentration with vaccine off-target antibody response. *Vaccine off-target antibody response is represented by the 1st PLS-DA component scores (y-axis) of the PLS-DA trained with the log-transformed normalized antibody signals from the protein array other than anti-CSP IgG as predictors and vaccination group as the outcome (see Figure 2 from main manuscript).*

Figure 1 is a replication of ***Figure 2*** from the main manuscript where CSP Ab levels (x-axis) were now measured by ELISA separately for the NANP repeat and C-Term regions. As noted, high vaccine off-target responses can only occur in RTS,S/AS01E vaccinated individuals (a necessary condition) but within vaccinated individuals a high off-target Ab response is not guaranteed (not a sufficient condition). When focusing on RTS,S/AS01E vaccinated individuals (green dots), a stronger correlation of the off-target Ab response score existed with anti-NANP IgG (ρ=0.48, CI [0.44-0.53], p< 2.2e-16), compared to the one with anti-C-Term IgG (ρ=0.11, CI [0.02, 0.19], p=0.014).

Comparing high against low off-target Ab responders using the dichotomous classification based on the seven most increased off-target Abs, we confirmed an association only with NANP IgG levels in the same direction. RTS,S/AS01E vaccinated individuals classified as high responders had IgG concentrations with a geometric mean 2.19 times higher, 95% CI [1.73, 2.75] (p=1.84e-10), than the low Ab off-target responders. In contrast, no significant difference was found for C-Term IgG levels (fold-difference of 1.31, CI [0.91, 1.32], p=0.84). Fold differences in geometric means were estimated by fitting a multiple regression with $\log_{10}$

IgG as the continuous outcome, the off-target Ab response group as the binary predictor of interest, and adjusting for age group and site.

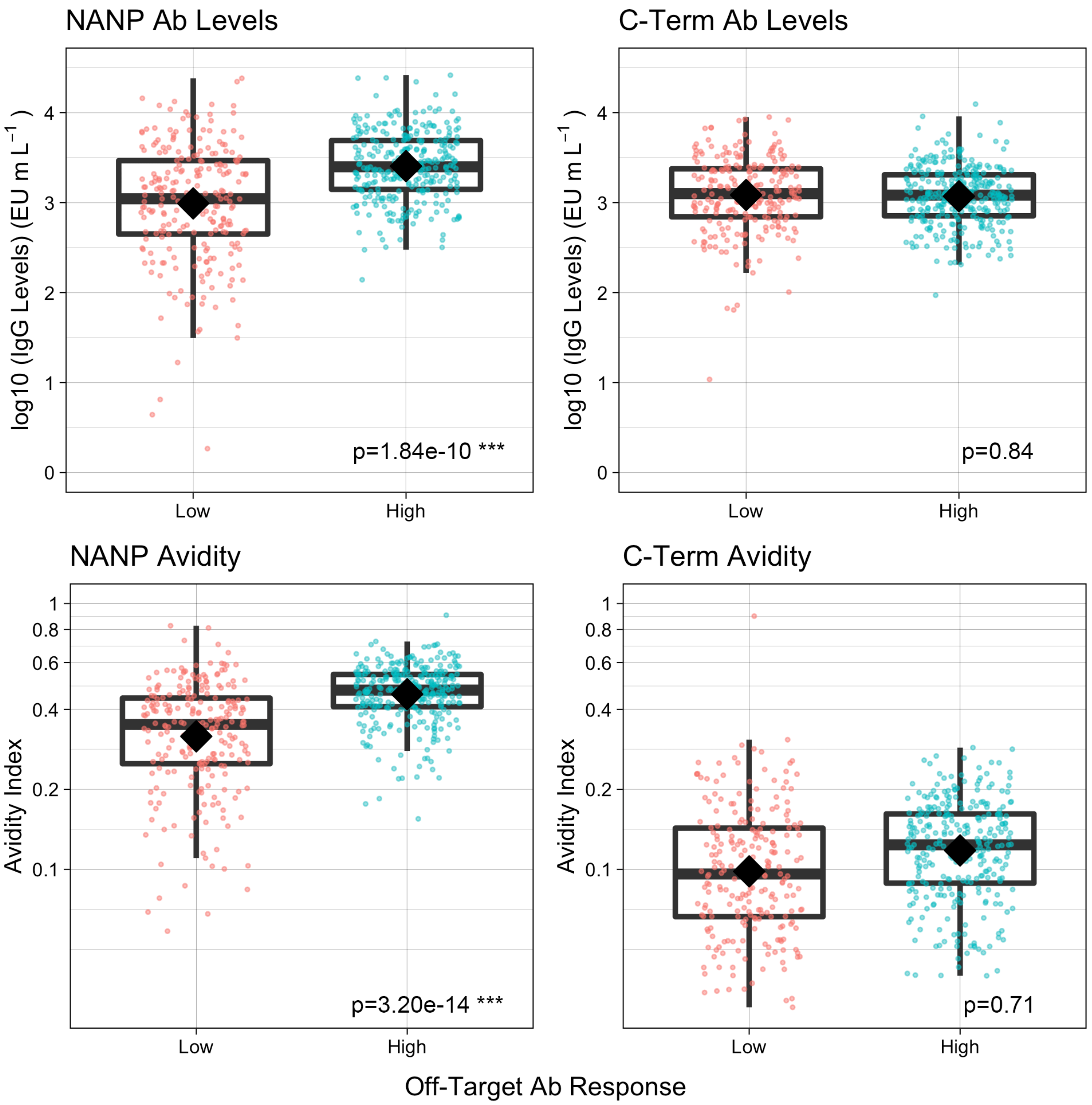

Figure 2. Association of NANP and C-Term CSP Ab levels and avidity with vaccine off-target Ab response group*. Boxplots illustrate the medians and the 25th and 75th quartiles, diamonds show the geometric mean, whiskers display the 1.5 interquartile ranges, and coloured dots are measured data. Significance of the geometric mean differences are calculated as t-tests on the regression coefficients in models adjusted by site and age group*

Additionally, we conducted the same comparisons using IgG avidity as outcome and also found an association with vaccine off-target Ab response for NANP (a 31% higher geometric mean Avidity Index in high off-target Ab responders, CI [22%, 42%], p=3.20e-14) but not for C-Term (2%, CI [-8%, 13%], p=0.71) (see *Figure 2*). Of note, this significant association of higher post-vaccination NANP IgG avidity in high off-target Ab responders remained when adjusted for NANP IgG levels (28% higher geometric mean Avidity Index, CI [20%, 37%]), considering that Ab avidity correlates with Ab levels (Dobaño et al., 2019).

These results indicate that off-target reactivity is related to the immunogenicity of the immunodominant NANP Abs rather than to that of the subdominant C-Term Abs. Furthermore, if anti-NANP IgGs cross-reacting with other non-vaccine *P. falciparum* antigens in some individuals explain off-target reactivity, then data suggest that these Abs do not bind with less affinity to their cognate NANP target and, therefore, this phenomenon may not be attributed to a potential lack of specificity and/or maturation.

References

1. Dobaño C, Sanz H, Sorgho H, et al. Concentration and avidity of antibodies to different circumsporozoite epitopes correlate with RTS,S/AS01E malaria vaccine efficacy. *Nat Commun*. 2019;10(1):2174. Published 2019 May 15. doi:10.1038/s41467-019-10195-z

Supplementary Material 5. Clinical malaria incidence ratios of High over Low off-target antibody responders estimated with and without lost-to-follow-up vaccinees

Table 1. Complete Case Analysis. Individuals who did not complete follow-up (6, 12 or 18 months after M3) were excluded from analyses. These analyses are those reported in the main manuscript

NOT ADJUSTED for anti-CSP antibody levels				
	Nº Included Vaccinees	IR	95 % CI	p-value
0 – 6 Months	1352	0.61	[0.50, 0.76]	8.83e-06 ***
6 – 12 Months	1322	0.77	[0.65, 0.91]	2.53e-03 **
12 – 18 Months	1285	0.78	[0.66, 0.93]	5.20e-03 **
**ADJUSTED for anti-CSP antibody levels**				
		IR	95 % CI	p
0 – 6 Months	1352	0.71	[0.57, 0.88]	1.82e-03 **
6 – 12 Months	1322	0.86	[0.72, 1.02]	0.075 ·
12 – 18 Months	1285	0.83	[0.69, 0.99]	0.035 *

Table 2. Incomplete Case Analysis. Individuals who did not complete follow-up (6, 12 or 18 months after M3) were included if followed for longer than 1 month after adjusting for their shorter time at risk in the offset of the negative binomial regression.

NOT ADJUSTED for anti-CSP antibody levels				
	Nº Included Vaccinees	IR	95 % CI	p-value
0 – 6 Months	1376	0.61	[0.49, 0.76]	6.96e-06 ***
6 – 12 Months	1352	0.78	[0.66, 0.92]	3.53e-03 **
12 – 18 Months	1322	0.76	[0.64, 0.91]	1.95e-03 **
**ADJUSTED for anti-CSP antibody levels**				
		IR	95 % CI	p-value
0 – 6 Months	1376	0.70	[0.56, 0.87]	1.21e-03 **
6 – 12 Months	1352	0.87	[0.73, 1.03]	0.10 ·
12 – 18 Months	1322	0.80	[0.67, 0.96]	0.014 *
